# Disparities in Obstetric, Neonatal, and Birth Outcomes Among Syrian Women Refugees and Jordanian Women

**DOI:** 10.3389/ijph.2023.1605645

**Published:** 2023-11-03

**Authors:** Tariq N. Al-Shatanawi, Yousef Khader, Nadin Abdel Razeq, Ahmed M. Khader, Mahmoud Alfaqih, Osama Alkouri, Mohammad Alyahya

**Affiliations:** ^1^ Department of Public Health and Community Medicine, Al-Balqa Applied University, Al-Salt, Jordan; ^2^ Department of Public Health, Jordan University of Science and Technology, Irbid, Jordan; ^3^ Department of Maternal and Child Health Nursing, School of Nursing, The University of Jordan, Amman, Jordan; ^4^ Faculty of Medicine, Jordan University of Science and Technology, Irbid, Jordan; ^5^ Department of Physiology and Biochemistry, Jordan University of Science and Technology, Irbid, Jordan; ^6^ Faculty of Nursing, Yarmouk University, Irbid, Jordan; ^7^ Department of Health Management and Policy, Jordan University of Science and Technology, Irbid, Jordan

**Keywords:** Jordan, mortality, Syrian refugee, pregnancy outcomes, neonates

## Abstract

**Objectives:** To compare obstetric and neonatal characteristics and birth outcomes between Syrian refugees and native women in Jordan.

**Methods:** We used the Jordan Stillbirths and Neonatal Deaths Surveillance System to extract sociodemographic and obstetric characteristics of the mothers and birth characteristics of newborns. Multivariate analysis was used to compare the characteristics of 26,139 Jordanian women (27,468 births) and 3,453 Syrian women refugees (3,638 births) who gave birth in five referral hospitals (May 2019 and December 2020).

**Results:** The proportions of low birthweight (14.1% vs. 11.8%, *p* < 0.001) and small for gestational age (12.0% vs. 10.0%, *p* < 0.001) newborns were significantly higher for those born to Syrian women compared to those born to Jordanian women. The stillbirth rate (15.1 vs. 9.9 per 1,000 births, *p* = 0.003), the neonatal death rate (21.2 vs. 13.2 per 1,000 live births, *p* < 0.001), and perinatal death rate (21.2 vs. 13.2 per 1,000 births, *p* < 0.001) were significantly higher for the Syrian births. After adjusting for sociodemographic and obstetric characteristics of women, only perinatal death was statistically significantly higher among Syrian babies compared to Jordanian babies (OR = 1.3, 95% CI: 1.1–1.7, *p* = 0.035).

**Conclusion:** Syrian refugee mothers had a significantly higher risk of adverse obstetric and neonatal outcomes including higher rate of perinatal death compared to Jordanian women.

## Introduction

Forced migration occurs globally for various reasons, including wars and political conflicts, economic circumstances, or environmental crises [1]. Women refugees of all nationalities are a particularly vulnerable group of displaced people. Having a higher reported prevalence of adverse maternal-neonatal outcomes than native women [2, 3] makes them a unique population in host communities in terms of needed humanitarian and healthcare services. Stillbirths, delayed arrival at the hospital for birth [4, 5], inappropriate weight for gestational age [2], low birthweight infants [6], and higher rates of neonatal intensive treatments for neonatal comorbidities [2] are a few adverse pregnancy outcomes that are commonly reported among women refugees. However, contemporary research and knowledge on women refugees’ health during pregnancy and pregnancy outcomes is limited in the international literature. Therefore, further understanding of how their health is compared to non-refugee counterparts is needed [3].

Since March 2011, Syria has been in a state of political instability that has resulted in about 13.5 million Syrians being forcibly displaced to escape the war, out of which 6.8 million sought refuge in their country [7]. The neighbouring country of Jordan has been the host of more than one million Syrian refugees [8], of which only 673,957 have registered with the United Nations High Commissioner for Refugees (UNHCR) as of February 2022 [9]. Women and girls constitute 49.6% of all registered refugees in Jordan, and 30% of them are above the age of 12 years [9]. Further, reports indicate that the majority (>85%) of registered Syrian refugees live outside camps, in urban, peri-urban, or rural regions of Jordan [9]. According to recent assessments of the needs of refugees for financial and non-financial services [10, 11], more than 86% of Syrian refugees live under the Jordanian poverty line [11] despite the substantial response by government and humanitarian partners.

In Jordan, healthcare services, including pregnancy and delivery services, are available to all registered refugees from all nationalities at the non-insured Jordanian rate at public health centres and governmental hospitals [12]. However, because most Syrian refugees are not insured and must pay health-related fees for hospitals and clinics, Syrian refugees identified cost as the main barrier to healthcare access [13]. The UNHCR supports healthcare services for all refugees. However, research has shown that Syrian women refugees in Jordan continue to have significant unmet needs and barriers to access, utilisation, and implementation of sexual and reproductive health (SRH) in comparison to their host community counterparts [14, 15].

There is limited research assessing the reproductive health status and service delivery among Syrian refugees in Jordan [14]. Currently, there is little understanding of the risks of adverse pregnancy outcomes among Syrian women refugees in Jordan and the extent to which disparities exist regarding pregnancy outcomes. The adverse maternal and neonatal pregnancy outcomes and disparities need to be addressed first, and their contributing factors should be systematically investigated to improve the maternal healthcare utilisation and health services provided to Syrian women refugees.

The Jordan Stillbirths and Neonatal Deaths Surveillance System (JSANDS) is an electronically secure surveillance system to register and disseminate reliable, comprehensive, individual-level data on stillbirths and neonatal deaths, the causes and the risk factors most commonly associated with perinatal mortality in Jordan [16–18]. The system record events for women prospectively after giving their births. The surveillance system automatically and accurately transfers the data on births, stillbirths, and neonatal deaths from five maternity hospitals to the Ministry of Health [16]. These hospitals are among the most significant and primary referral hospitals in Jordan that cover most births and deaths in all regions in the north, east, and south of Jordan. Khader, Alyahya [16] examined the JSANDS attributes, including its data quality and sensitivity. The system users rated the usefulness of JSANDS as excellent (percentage score = 85.6%). The overall acceptability (percentage score = 82.3%), flexibility (percentage score = 80.2%), stability (percentage score = 80.0%), and representativeness (percentage score = 86.6%) were also rated excellent. All variables in JSANDS had complete data with no missing values for clinical variables because these data are entered by nurses who were trained to enter data completely and because the system has automated quality checks. The overall simplicity was scored well (percentage score = 75.4%) [16].

The purpose of this study is to explore the adverse birth outcomes among Syrian women refugees and Jordanian women using data from the JSANDS. Specifically, in this study we identified disparities of the obstetric and neonatal characteristics and birth outcomes between Syrian and Jordanian women, and explored the factors determining adverse neonatal birth outcomes among Syrian women in Jordan.

## Methods

### Study Design

All births, stillbirths, and neonatal deaths in five hospitals from May 2019 to December 2020 were completely registered in JSANDS. The five hospitals, including one university teaching hospital, three public hospitals, and one private hospital, were selected for pilot testing of the JSANDS. The criteria for the selection of hospitals and JSANDS usability and functionality were described in previous studies [17, 19, 20]. The sociodemographic and obstetric characteristics of Jordanian and Syrian women and their birth outcomes were retrieved from JSANDS and compared.

### Variables

The extracted data consisted of sociodemographic and obstetric characteristics of the mothers, including age, education, employment status, income, gestational age, mode of delivery, and multiplicity. Other data included information on neonatal characteristics and birth outcomes, such as birthweight and status (born alive, stillbirth, and neonatal death).

### Definitions

Birthweight was categorised into low (1,500 g–<2,500 g), very low (<1,500 g–1,000 g), and extremely low birthweight (<1,000 g) [21]. The gestational age was categorised into extremely preterm (<28 weeks), very preterm (28–<32 weeks), and moderate or late preterm (32–<37 completed weeks of gestation) [22]. The definition of stillbirths and neonatal deaths used in this study was based on the international standards set by the World Health Organization [23]. Neonatal mortality was defined as any death that happened at or after 24 gestational weeks within the first 28 days of life. The neonatal mortality rate was calculated as the number of neonatal deaths per 1,000 live births (LB). Stillbirth was defined as any foetal death that occurred at or after 24 gestational weeks. Stillbirths were categorised as antepartum (deaths occurring prior to labour) and intrapartum (deaths that occur after the onset of labour but prior to birth). The stillbirth rate was calculated as the number of stillbirths per 1,000 total births. The foetal weight percentile was determined based on birthweight and foetal age. Foetal growth was categorised as small for gestational age (SGA) if <10th percentile, appropriate for gestation if between 10th and 90th percentile, or large for gestation if >90th percentile.

### Ethical Considerations

This study was performed in line with the principles of the Declaration of Helsinki. The study was ethically approved by Jordan University of Science and Technology Institutional Review Board (IRB) (Ethical approval number 20170033) to conduct the study at King Abdulla University Hospital, Jordan Ministry of Health Review Committee to conduct the study in the three public hospitals, and the Irbid Speciality Hospital (private hospital) Ethical Committee. Permission from the Ministry of Health was received to access the data and use it for research purposes. To ensure the confidentiality of the data, the data were exported without identifying information, such as the name or phone number.

### Data Analysis

Data were described and analysed using IBM SPSS version 24 (IBM Corp. Released 2016. IBM SPSS Statistics for Windows, Version 24.0. Armonk, NY: IBM Corp.). Percentages were used for categorical data. The sociodemographic and obstetric characteristics of Jordanian and Syrian women and their birth outcomes were compared and analysed using the Chi-square test. Multivariate analysis using binary logistic regression was performed to compare the birth outcomes of Jordanian and Syrian women after adjusting for women’s characteristics. A *p*-value of less than 0.05 was considered statistically significant.

## Results

### Women’s Characteristics

This study included a total of 26,139 Jordanian women (27,468 births) and 3,453 Syrian women refugees (3,638 births). Of the total Jordanian and Syrian births, 271 and 55 were stillbirths, and 360 and 76 died before 28 days of life, respectively. The sociodemographic and obstetric characteristics of Jordanian and Syrian women are shown and compared in Table 1.

**TABLE 1:** The sociodemographic and obstetric characteristics of Jordanian and Syrian women (Jordan, May 2019 and December 2020).

	Nationality				*p*-value
	Jordanian		Syrian refugees		
	*n*	%	*n*	%	
Mother’s age (years)					<0.001
<20	982	3.8	423	12.3	
20–35	20,590	78.8	2,590	75.0	
>35	4,567	17.5	440	12.7	
Mother’s occupation					<0.001
Housewife	23,252	89.0	3,397	98.4	
Employed	2,887	11.0	56	1.6	
Mother’s education					<0.001
Diploma or less	16,713	68.6	2,290	94.7	
Bachelor or higher	7,660	31.4	129	5.3	
Income (JD)					<0.001
≤500	19,901	80.5	3,087	96.7	
>500	4,811	19.5	105	3.3	
Type of hospital at birth					<0.001
Public hospital	17,376	66.5	2,376	68.8	
Private hospital	4,872	18.6	1,036	30.0	
Teaching hospital	3,891	14.9	41	1.2	
Multiplicity					0.022
Singleton	24,916	95.3	3,299	95.5	
Twin	1,140	4.4	134	3.9	
Triplet or more	83	0.3	20	0.6	
Parity					<0.001
Nulliparity	842	3.2	92	2.7	
Low multiparity (parity 1–3)	17,414	66.6	1,992	57.7	
Grand multipara (parity ≥4)	7,883	30.2	1,369	39.6	
History of neonatal death	447	1.7	85	2.5	0.002
History of stillbirth	4,935	18.9	652	18.9	0.997
Gestational age					0.137
Full-term	23,761	90.9	3,112	90.1	
Preterm	2,378	9.1	341	9.9	
Mode of delivery					<0.001
Vaginal	13,017	49.8	1,772	51.3	
Planned CS	6,982	26.7	1,060	30.7	
Emergency CS	5,957	22.8	604	17.5	
Instrumental	183	0.7	17	0.5	

Syrian women were statistically significantly younger than Jordanian women (12% and 4% under age 20 years, respectively, *p* < 0.001). The mean (SD) age was 29.3 (6.0) year for Jordanian mothers and 27.3 (6.7) year for Syrian women. Only 11.0% and 1.6% of Jordanian and Syrian women were employed, respectively (*p* < 0.001). Jordanian women had a higher level of education than Syrian women (31.4% and 5.3% of Jordan and Syrian women had bachelor’s or higher degrees, respectively). Moreover, monthly income was statistically significantly higher for Jordanian women than for Syrian women. Jordanian and Syrian women differed statistically significantly in terms of multiplicity rate, parity, and mode of delivery. The mean (SD) parity was 2.8 (1.7) for Jordanian mothers and 3.3 (2.0) year for Syrian women.

### Birth Outcomes

Table 2 shows the birth outcomes for Jordanian and Syrian newborns. In comparison to the babies born to Jordanian women, the proportion of babies born with low weight was statistically significantly higher for Syrian women (11.8% vs. 14.1%, *p* < 0.001). About 10.0% and 12.0% of babies born to Jordanian and Syrian women were SGA (*p* < 0.001), respectively.

**TABLE 2:** Birth outcomes for Jordanian and Syrian newborns (Jordan, May 2019 and December 2020).

	Nationality				*p*-value
	Jordanian		Syrian refugees		
	*n*	%	*n*	%	
Preterm birth	2,874	10.5	415	11.4	0.082
Low birthweight	3,236	11.8	512	14.1	<0.001
Small for GA	2,742	10.0	437	12.0	<0.001
Stillbirth rate (per 1,000 total births)	271	9.9	55	15.1	0.003
Neonatal death rate (per 1,000 live births)	360	13.2	76	21.2	<0.001
Perinatal death rate (per 1,000 total births)	549	20.0	116	31.9	<0.001

**FIGURE 1:**
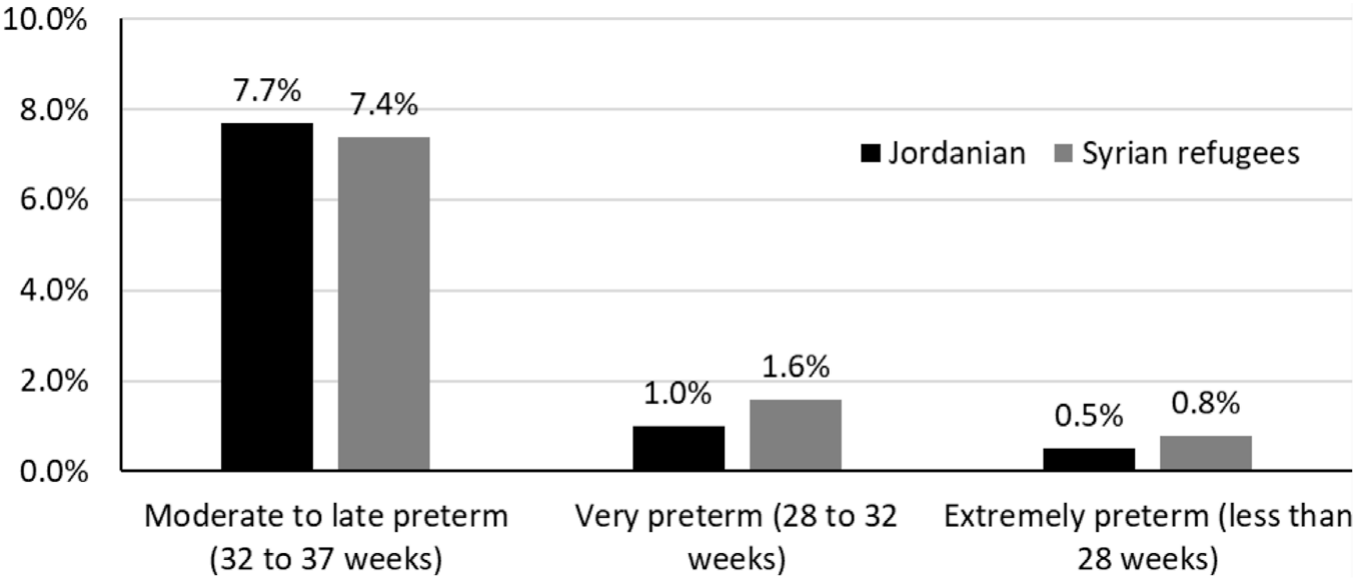
The distribution of preterm delivery among Jordanian and Syrian women (Jordan, May 2019 and December 2020).

**FIGURE 2:**
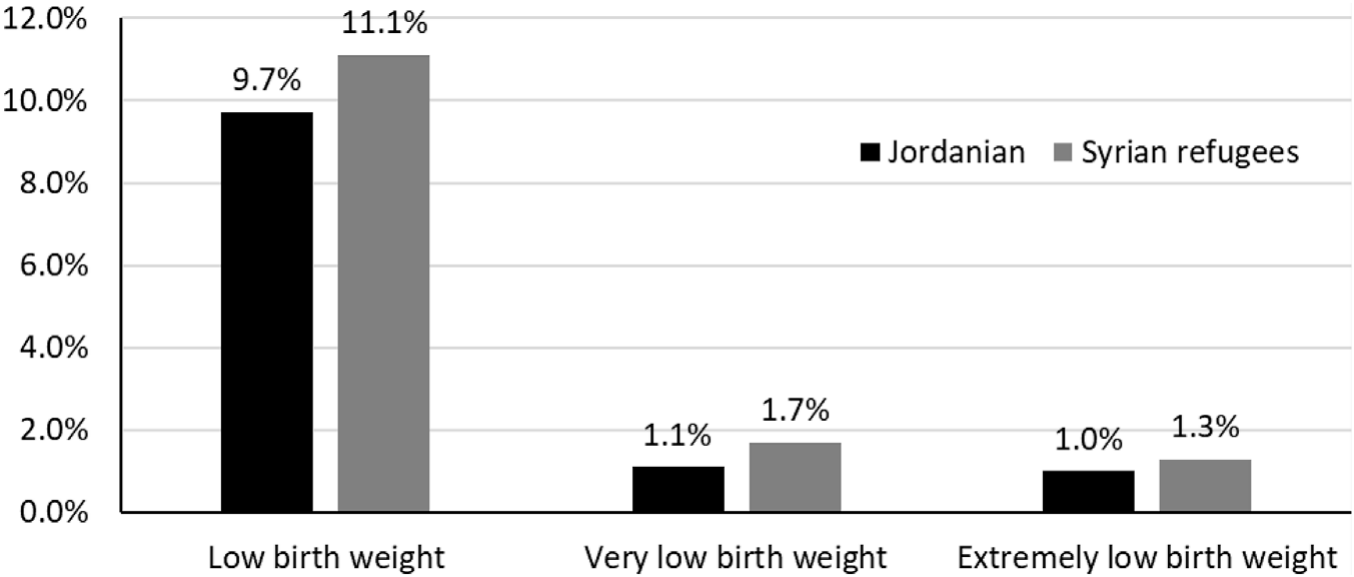
The distribution of low-birth-weight babies born to Jordanian and Syrian women (Jordan, May 2019 and December 2020).

**FIGURE 3:**
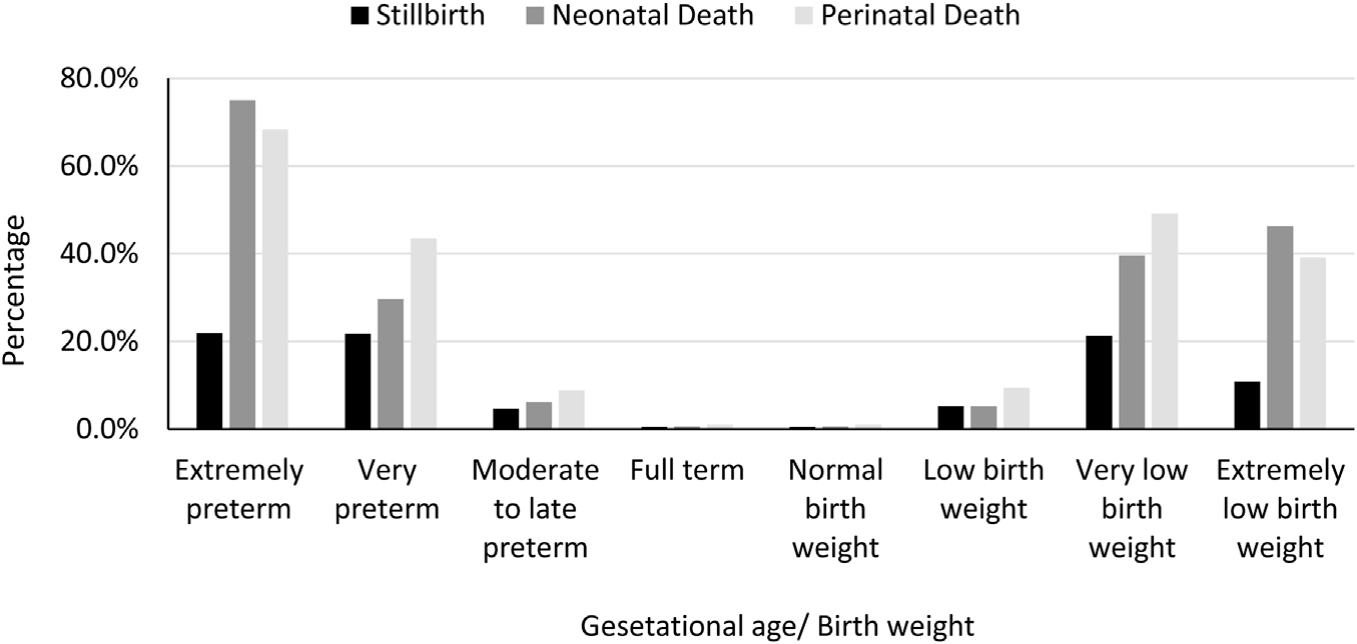
The distribution of stillbirths, neonatal deaths and perinatal deaths for babies born to Syrian women according to gestational age and birth weight (Jordan, May 2019 and December 2020).

The distribution of preterm delivery and birthweight for Syrian and Jordanian babies is shown in Figures 1, 2, respectively. The stillbirth rate (15.1 vs. 9.9 per 1,000 total births, *p* = 0.003), the neonatal death rate (21.2 vs. 13.2 per 1,000 live births, *p* < 0.001), and perinatal death rate (21.2 vs. 13.2 per 1,000 total births, *p* < 0.001) were statistically significantly higher among Syrian births compared to Jordanian births. The distribution of stillbirths, neonatal deaths and perinatal deaths for babies born to Syrian women according to gestational age and birth weight are shown in Figure 3. The vast majority of deaths occurred among babies born prematurely or with low birth weight.

### Multivariate Analysis

In the bivariate analysis, the proportion of babies born with low birthweight or SGA, stillbirth rate, neonatal death rate, and perinatal death rate was statistically significantly higher among babies born to Syrian women than those born to Jordanian women. After adjusting for sociodemographic and obstetric characteristics of women (Table 3), only perinatal death was statistically significantly higher among Syrian babies compared to Jordanian babies (OR = 1.3, 95% CI: 1.1–1.7, *p* = 0.035).

**TABLE 3:** Multivariate analysis of differences in birth outcomes for Jordanian newborns versus Syrian newborns (Jordan, May 2019 and December 2020).

	Univariate analysis				Multivariate analysis*			
	OR	95% confidence interval		*p*-value	OR	95% confidence interval		*p*-value
Preterm birth	1.1	1.0	1.2	0.082	1.0	0.9	1.2	0.936
Low birthweight	1.2	1.1	1.4	<0.001	1.1	0.9	1.2	0.243
Small for GA	1.2	1.1	1.4	<0.001	1.1	1.0	1.3	0.081
Stillbirth	1.5	1.2	2.1	0.004	1.3	0.9	1.9	0.181
Neonatal death	1.6	1.3	2.1	<0.001	1.2	0.9	1.7	0.271
Perinatal death	1.6	1.3	2.0	<0.001	1.3	1.1	1.7	0.035

*Adjusted for mother’s age, employment status, education, income, baby gender, multiplicity, parity, history of neonatal death, and history of stillbirth.

## Discussion

This study is the first to systematically assess and compare wide-ranging birth outcomes and disparities among Syrian women in the host community of Jordan. Using comprehensive multicentre data, our analysis specifically provided information about the discrepancies in LBW (<2,500 g), SGA, preterm delivery, birthweight, stillbirth, and perinatal death rate among Syrian and Jordanian women’s births.

Our findings indicate remarkable disparities in pregnancy outcomes among the Syrian women refugees in the host communities of Jordan. Compared to the Jordanian women, the adverse pregnancy outcomes were remarkably higher among the Syrian women refugees, a gravely concerning finding that resonated with a previous retrospective study conducted at two governmental hospitals in northern Jordan [24]. The previous study documented a significant inequality in terms of the rate of caesarean section, anaemia, and lower neonates’ APGAR scores and birthweight among Syrian women deliveries compared to those from Jordan [24]. When summed together, these findings indicate a priority for more effective and tailored maternal-newborns healthcare services directed toward the refugee mothers in Jordan. Transformation is required in the current healthcare policies and interventions implemented in Jordan to improve women refugee’s access to and quality of antenatal, birth, and postnatal healthcare services.

Neonatal health outcomes, in general, have improved considerably in Jordan, as indicated by a slow but progressive decline in neonatal death rates over the past 20 years [25]. According to international reports, Jordan’s overall neonatal mortality rate is recorded at 9 per 1,000 LB [25], and above 14 per 1,000 LB, according to research-based information conducted nationally [19, 26]. Comparing our findings to these national reports, the neonatal and perinatal death rates recorded among Syrian births are remarkably higher than those reported nationally and internationally [19, 25, 26]. Adverse pregnancy outcomes are significantly high among Syrian women refugees on all measured parameters in this study. Our analysis showed that the overall birth outcomes among the Syrian women refugees in Jordan are poor and create a threat to achieving Jordan’s national and international development sustainability goals [27, 28].

Our findings regarding certain birth outcomes (e.g., weight at birth, SGA, low birthweight) were in agreement with those that have been reported among displaced Syrian women in a few comparable studies from Lebanon [6, 29] and Turkey [2, 5, 30]. However, in opposition to our findings, numerous studies from Turkey demonstrated no disparities affecting Syrian women concerning caesarean birth rates [2, 30–33], preterm delivery [30, 32, 33], stillbirth [5, 34], abortion [32, 33], LBW (<2,500 g) [33, 35], and small for gestational birthweight [5]. These discrepancies could suggest that health policies and interventions that facilitate women refugees’ access to and the provision of quality maternal and neonatal services could positively reduce the prevalence and disparities of adverse pregnancy outcomes among women refugees. An international conclusion should be drawn with caution and should be interpreted within its terrestrial context, considering the variations in the quality of healthcare services across countries.

Our findings also showed that other disparities also existed in the sociodemographic characteristics that are related to birth outcomes of the mothers from Syria compared to those from Jordan, specifically in terms of younger age, income, unemployment, and educational attainment. The results showed that the difference can be attributed to the background variables among the Jordanian and Syrian women. After controlling for these factors, the difference between the two groups of women seems to become not significant. These sociodemographic discrepancies could have contributed to worsening pregnancy outcomes and widening the healthcare disparity gap among the Syrian women refugees in Jordan. Low socioeconomic state [6], early-age/adolescent pregnancy [35], infrequent antenatal visits [6, 32, 34, 35], and the late arrival to skilled maternal care at birth [5, 24, 35] are contributing factors to adverse pregnancy outcomes that were prevailing among women refugees from Syria in other countries. The adverse birth outcomes are multifactorial, and this should be considered when designing and delivering care services to women refugees in the host countries.

Adolescent pregnancy, in particular, has been consistently observed to be prevalent among Syrian displaced women compared to native women in host countries [2, 35–38], which is in accordance with our comparison in Jordan. Adolescent pregnancy is one crucial factor associated with adverse birth outcomes worldwide [39, 40], which is similar to the case of Jordan [41]. The socioeconomic and healthcare needs, risks and pregnancy outcomes of Syrian adolescents and young women should receive further attention through humanitarian field assessments and research work. Our findings sheds light on the importance of designing healthcare policies and services to provide additional support for the vulnerable groups of women refugees.

### Strengths and Limitations

Retrospective studies on expositing data are efficient means of gathering data in public health studies; however, researchers are limited with the nature and amount of the data excited. Although the current analysis was conducted in five leading referral hospitals in Jordan, the findings might only be transferred to some Jordanian women and Syrian refugees that might receive care outside these hospitals in the country. Despite this limitation, this study is the first multicentre study in Jordan to compare wide-ranging sociodemographic and birth outcomes between displaced Syrian refugees and Jordanian women. This study pave the way for future improvement in obstetric, neonatal, and birth outcomes and there related surveillance system.

### Conclusion

Syrian refugee mothers had higher rates of adverse birth outcomes and inequalities than women in Jordan’s host communities. Ongoing evaluation of pregnancy outcomes is essential to enhance our understanding of the healthcare disparities among refugees in the host communities. However, it is more important to foster a comprehensive understanding of the multilevel contributing factors to help develop strategies on micro and macro levels for controlling these factors. Our findings suggest a healthcare priority for better improvement in the policies and provision of maternal-neonatal care services to women refugees in Jordan. Healthcare policies and services may be designed to provide additional support for these vulnerable groups of women refugees.
